# A Portable Wearable Inertial System for Rehabilitation Monitoring and Evaluation of Patients With Total Knee Replacement

**DOI:** 10.3389/fnbot.2022.836184

**Published:** 2022-03-23

**Authors:** Nan Lou, Yanan Diao, Qiangqiang Chen, Yunkun Ning, Gaoqiang Li, Shengyun Liang, Guanglin Li, Guoru Zhao

**Affiliations:** ^1^Department of Orthopedics, University of Hong Kong–Shenzhen Hospital, Shenzhen, China; ^2^CAS Key Laboratory of Human-Machine Intelligence-Synergy Systems, Research Center for Neural Engineering, Shenzhen Institute of Advanced Technology, Chinese Academy of Sciences, Shenzhen, China; ^3^Shenzhen College of Advanced Technology, University of Chinese Academy of Sciences, Shenzhen, China; ^4^Guangdong-Hong Kong-Macao Joint Laboratory of Human-Machine Intelligence-Synergy Systems, Shenzhen Institute of Advanced Technology, Chinese Academy of Sciences, Shenzhen, China

**Keywords:** total knee replacement (TKR), rehabilitation progress, wearable inertial unit, monitoring and evaluation, knee range of motion (ROM)

## Abstract

Knee osteoarthritis is a degenerative disease, which greatly affects the daily life of patients. Total knee replacement (TKR) is the most common method to treat knee joint disorders and relieve knee pain. Postoperative rehabilitation exercise is the key to restore knee joint function. However, there is a lack of a portable equipment for monitoring knee joint activity and a systematic assessment scheme. We have developed a portable rehabilitation monitoring and evaluation system based on the wearable inertial unit to estimate the knee range of motion (ROM). Ten TKR patients and ten healthy adults are recruited for the experiment, then the system performance is verified by professional rehabilitation equipment Baltimore Therapeutic Equipment (BTE) Primus RS. The average absolute difference between the knee ROM and BTE Primus RS of healthy subjects and patients ranges from 0.16° to 4.94°. In addition, the knee ROM of flexion-extension and gait activity between healthy subjects and patients showed significant differences. The proposed system is reliable and effective in monitoring and evaluating the rehabilitation progress of patients. The system proposed in this work is expected to be used for long-term effective supervision of patients in clinical and dwelling environments.

## Introduction

Knee osteoarthritis (KOA), as a degenerative disease, is typically the result of wear and tear and gradual loss of articular cartilage caused by age, obesity, trauma, etc., (Hsu and Siwiec, 2021). Previous studies showed that among adults over 60 years old in the United States, the prevalence of symptomatic KOA is about 10% in men and about 13% in women (Zhang and Jordan, 2010). KOA greatly affects the activities of daily living (ADL) and quality, and total knee replacement (TKR) is the main method to treat knee joint diseases and relieve knee pain (Gauchard et al., 2010; Skou et al., 2018). The artificial joint prosthesis is implanted through surgical technology to replace the diseased joint, relieve joint pain and restore joint function. In the United States, approximately 1.3 million KOA patients undergo TKR operations each year, and the operation rate is comparable to many European countries. In China, nearly 400,000 cases of TKR were reported in 2019, and the data is rising rapidly (Wang et al., 2019).

Physical therapy and rehabilitation training after TKR surgery is an important step for knee muscle strengthening and functional recovery. Many clinical cases have been reported in which patients have undergone appropriate surgical operations and failed to follow the training tasks assigned by the physical therapist after returning home, which ultimately led to poor functional recovery (Kontadakis et al., 2018). Therefore, the monitoring and evaluation of rehabilitation progress after TKR surgery is a clinical issue of concern for orthopedic surgeons (Artz et al., 2015). Naylor et al. found that early knee range of motion (ROM) can be used as an indicator to predict distant outcomes and provide favorable evidence for knee ROM required for clinical discharge (Naylor et al., 2012). Michael et al. pointed out in the paper that knee ROM and physical performance are the main results after TKR, and also powerful predictors of postoperative diagnosis (Bade et al., 2014). The knee ROM is not only a key parameter for evaluating the progress of knee function recovery but also an important measure of patient satisfaction with surgery (Ebert et al., 2014). Studies have shown that the knee ROM required to perform ADL is at least 100°. Specifically, knees need to be bent at 83° to ascend stairs, 90° to 100° to descend stairs, 93° to 105° to rise from a chair, and 115° to squat and kneel (Winemaker et al., 2012; Issa et al., 2015).

The current limb motion tracking technology mainly includes goniometers, optical systems, and inertial sensing systems (Kontadakis et al., 2018). As a traditional motion tracking method, goniometers are widely used in human joint motion measurement. Although it has the advantages of good physical adaptability and clinical convenience, it will also cause measurement deviation due to subjective judgment (Wang et al., 2011). The optical-based motion tracking system consists of a camera, reflection tracking markers, feature extraction technology, and imaging processing technology, with high precision. The disadvantage is that the system may be locally limited and expensive, and difficult to continuously monitor patients at home (Chiang et al., 2017). The inertial sensing system can capture all six degrees of freedom of the human body in real-time and has the advantages of low cost, portability, and freedom from time-space constraints (Takeda et al., 2009; Zihajehzadeh and Park, 2017; Wang et al., 2018). Therefore, this research uses wearable inertial sensors for data collection and data analysis to monitor the recovery progress of knee ROM in patients with TKR.

Many studies have developed their analysis methods using different combinations of wearable sensors to monitor and estimate knee ROM (Bakhshi et al., 2011; Seel et al., 2014; Feldhege et al., 2015; Ajdaroski et al., 2020). Ajdaroski et al. verified the ability of a single inertial measurement unit (IMU) to accurately measure the angle of the knee joint during dynamic motion. The results show that the IMU performs quite well under certain conditions, while the accuracy is low under some conditions (Ajdaroski et al., 2020). Feldhege et al. developed a novel inertial sensor system for walking behavior and joint motion measurement in daily environments. The results proved that the wearable sensor system showed high effectiveness for behavior classification and knee angle measurement in a laboratory environment (Feldhege et al., 2015). In principle, at least two IMUs need to be worn on the body, and then calculate the ROM with the knee joint as the fulcrum. For example, Seel et al. compared the method based on two wearable IMUs with an optical 3D motion capture system, and the results showed that the root means a square error of the knee flexion-extension angle was less than 3° (Seel et al., 2014). Bakhshi used two IMUs mounted on the thigh and shank to estimate the ROM, and the average error range in various tests was 0.08° to 3.06° (Bakhshi et al., 2011).

Although some studies have estimated the knee ROM in patients with TKR, the research focusing on monitoring the ROM of the knee joint is relatively insufficient. In addition, there are still some shortcomings in the system verification and estimation methods, which limit the real home application of the wearable system. Bell et al. (2019) verified the accuracy of the IMU-based measurement system to assess knee ROM, but the tested population consisted entirely of young and healthy volunteers, which may prevent the results from being directly translated into elderly patients. Huang et al. (2020) evaluated the knee ROM of patients with TKR based on a sensor system, but only static flexion and extension activities were performed, and there was a lack of dynamic monitoring such as gait activities. In the ROM estimation, since the IMU is installed on the irregular contour of the human body, the artifacts that accompany strenuous exercise will affect the results (Ajdaroski et al., 2020). Chiang et al. reported that when clinically estimating the TKR of a patient's knee, the sensor's zero-drift problem will have an impact on accuracy and is contrary to long-term home use (Chiang et al., 2017).

Based on the deficiencies in the rehabilitation assessment and monitoring of knee ROM after TKR, this research mainly focuses on three goals: (1) Develop a wearable hardware and software sensor system to facilitate patients' home rehabilitation monitoring; (2) Design a comprehensive experimental program involving static and dynamic flexion and extension, and verify it in TKR patients and healthy adults; (3) Propose a simple and accurate ROM estimation algorithm, which can effectively solve the problem of sensor zero drift and motion artifacts. In general, we hope that the developed system can be used on patients to provide monitoring and evaluation for exercise after TKR and help them restore knee joint function as soon as possible.

## Methods

**Figure 1:**
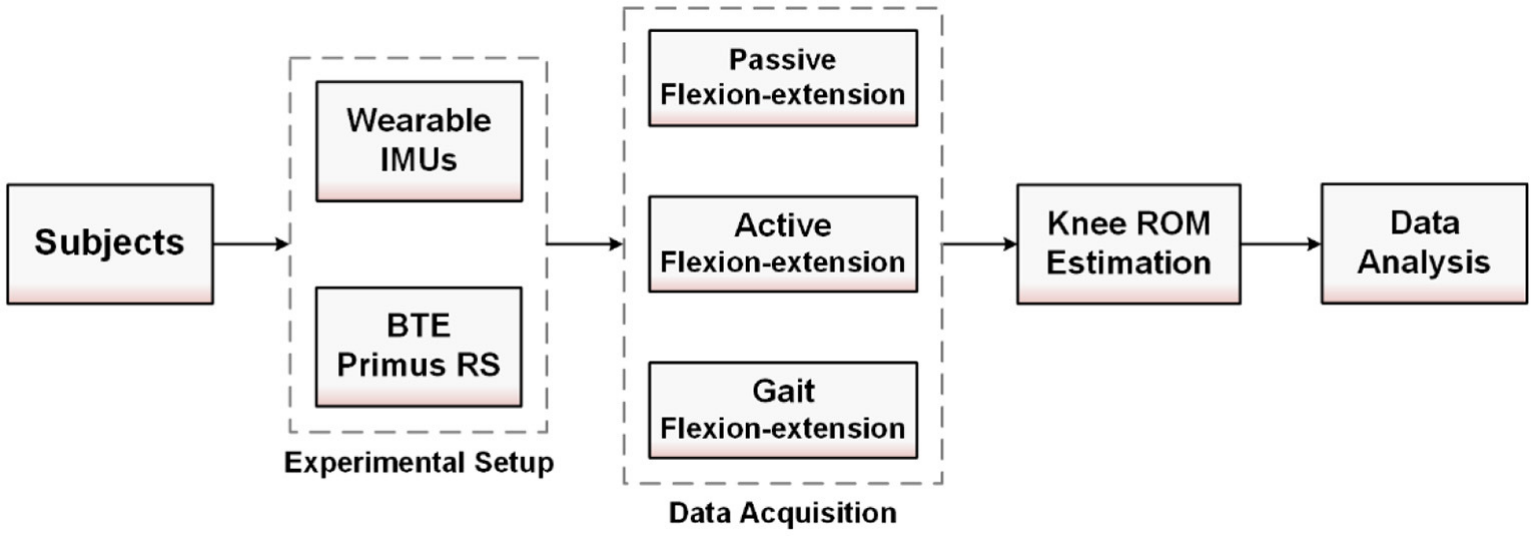
The flowchart and objectives of this experiment.

The flowchart of this research is shown in Figure 1. According to the flowchart, we divide this section into four subsections. Section Experimental Setup mainly introduces the selection criteria of patients and healthy adults, as well as the setting of experimental equipment; Section Data Acquisition describes the data collection process of the three flexion-extension activities; Section Knee ROM Estimation introduces the estimation process of knee ROM based on the equivalent model of flexion-extension activity; The last Section Data Analysis describes the data analysis objectives, including TKR patients and healthy subjects, replacement and non-replacement knee ROM. The detailed introduction of each section is described as follows.

### Experimental Setup

Twenty subjects (10 TKR patients and 10 healthy adults) are recruited to participate in this experiment. All patients are from the University of Hong Kong-Shenzhen Hospital, and the inclusion criteria are as follows: (1) Patients have undergone TKR due to osteoarthritis; (2) The recovery time for all patients from surgery is about 1 week; (3) Patients are between 60 and 80 years old; (4) Patients are not accompanied by severe osteoporosis; (5) Patients can walk with a walker or walk independently; (6) Patient are conscious and can complete all experiments. The healthy subjects are students from the laboratory, and with the age ranges from 20 to 35 years old. There are differences between healthy participants and patient ages, and we hope that the proposed algorithm has good generalization ability under different age groups and participants. Table 1 shows the cohort characteristics of the participants. Before the experiment, all participants sign an informed consent form, and all experiments involving human subjects have been approved by the Medical Research Ethics Committee of the Hong Kong University Shenzhen Hospital [No. Lun (2019) 175].

**Table 1:** Demographic characteristics of TKR patients and healthy adults.

**Cohort information**	**Mean ± Standard deviation**			
	**TKR patients**		**Healthy adults**	
Gender	Female	Male	Female	Male
Number	7	3	7	3
Age (years)	73.9 ± 6.36	71.3 ± 7.51	23.0 ± 2.58	26.7 ± 5.51
Height (cm)	158.7 ± 2.69	165.3 ± 1.53	160.6 ± 3.55	175.7 ± 4.73
Weight (kg)	64.9 ± 6.59	60.7 ± 1.15	50.9 ± 3.08	67.8 ± 4.07

**Figure 2:**
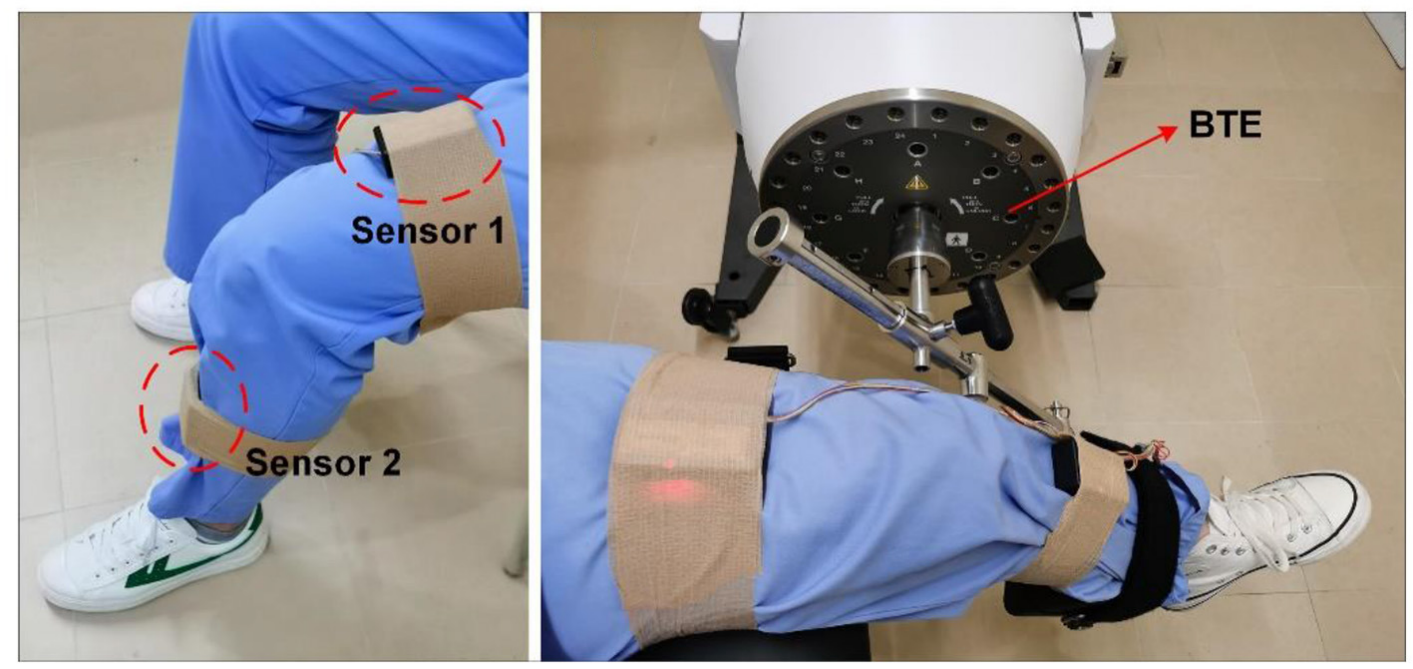
Inertial measurement units and BTE rehabilitation equipment.

The activity data is acquired by two wearable IMUs mounted on the thigh and shank, as shown in Figure 2. The sensor node consists of an STM32F407 microcontroller (STMicro electronics, Geneva, Switzerland), an MPU9250 accelerometer, and a gyroscope module (TDK InvenSense, San Jose, CA, USA), an Arduino Bluetooth module, and a lithium battery (300 mAh) (Diao et al., 2021). The size of the sensor node is 56.5 × 37.5 × 15.5 mm^3^, weighs about 30 g, and the sampling frequency is set to 100 Hz. The range of accelerometer and gyroscope are ±156.5 m/s^2^ (16 g) and ±34.9 rad/s (2000°/s), respectively. During data collection, the two sensor nodes are kept on the sagittal plane of the thigh and the shank.

Baltimore Therapeutic Equipment (BTE) Primus RS is advanced equipment integrating assessment, rehabilitation, and training, which is widely used in hospitals, rehabilitation centers, etc. (Suda et al., 2017; Torpel et al., 2017). In this experiment, BTE Primus RS is used as the standard for evaluating the knee ROM of patients. During the swing of the robotic arm, the angular velocity of the equipment can be manually adjusted to meet the needs of different subjects. The patient's shank is tied to the arm of BTE Primus RS, and the thigh is fixed on the chair so that accurate ROM could be recorded (Figure 2). Generally speaking, the smaller the difference between the estimated ROM and BTE Primus RS, the higher the accuracy of the proposed sensor system.

### Data Acquisition

**Figure 3:**
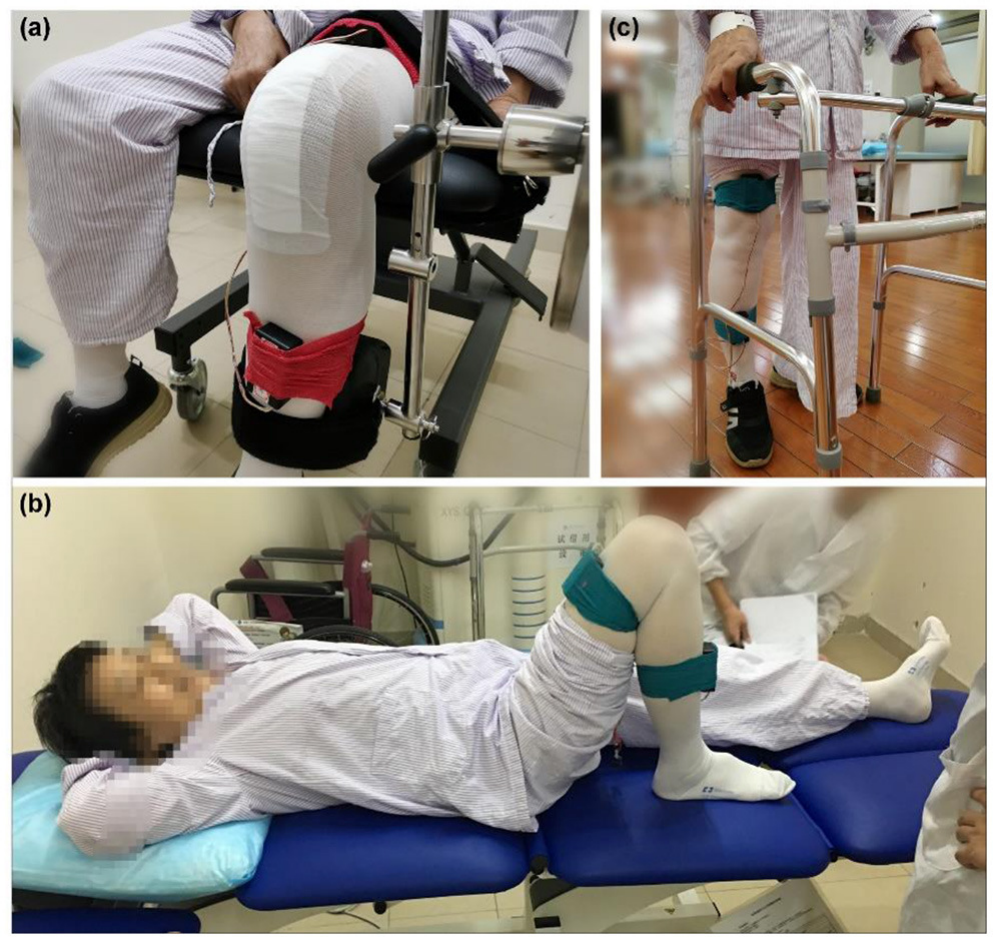
Three flexion-extension activities data collection. **(a)** Passive flexion-extension: Participants do not need to exert any force, and swing activities driven by BTE Primus RS. **(b)** Active flexion-extension: Participants need to complete the flexion-extension activity independently, and the maximum ROM is recorded. **(c)** Gait flexion-extension: The knee ROM of the participant during free walking is recorded.

To achieve accurate monitoring and evaluation during the rehabilitation of TKR patients, we have constructed a systematic experimental scheme to collect substantial clinical data. Experimental items include active and passive knee flexion-extension, gait activities, etc. Before data acquisition, the patients will be instructed and tested accordingly, and the BTE Primus RS equipment will be operated by a professional physical therapist. The detailed experimental procedure is described as follows (Figure 3).

(1) Passive Flexion-extension: Participants wear sensor IMUs, and are then asked to perform a flexion experiment driven by BTE Primus RS, as shown in Figure 3a. Considering the potential safety hazards for patients, three angular velocities are set at 15°/s, 30°/s, and 45°/s, rather than larger speeds. The test is repeated twice for each angular velocity, and the duration of each time is the 30s. The initial and final position of the flexion will be set, depending on the patient's tolerance. To ensure uniformity, all experiments of healthy adults are consistent with patients.

(2) Active Flexion-extension: Participants are required to lie flat on the bed with their arms under the head. Then slide one foot along the plane of the bed, from the far end to the proximal end, until it is unable to bend or painful, as shown in Figure 3b. The flexion-extension activities (twice on the left and right side, respectively) are repeated 3 times for each group of experiments.

(3) Gait flexion-extension: This test requires participants to walk 10 meters in a straight line, maintaining their rhythm. During the whole walking, the patients are uniformly asked to use a walking aid to prevent falls, as shown in Figure 3c. The gait activities are repeated twice for each participant.

### Knee ROM Estimation

To obtain an accurate knee ROM, the attitude angles of the two IMUs need to be calculated separately. The nine-axis IMU used in this research includes a three-axis accelerometer, a three-axis gyroscope, and an electronic compass. Although the gyroscope has high accuracy in a short time, due to drift and integral calculation, the long-term calculated attitude angle will produce cumulative errors. In contrast, accelerometers are easily affected by noise in a short time, but they have stable measurement accuracy for a long time (Tadano et al., 2016). In this study, the angle information obtained from the accelerometer was used to calibrate the gyroscope angle information to obtain accurate roll and pitch angles.

**Figure 4:**
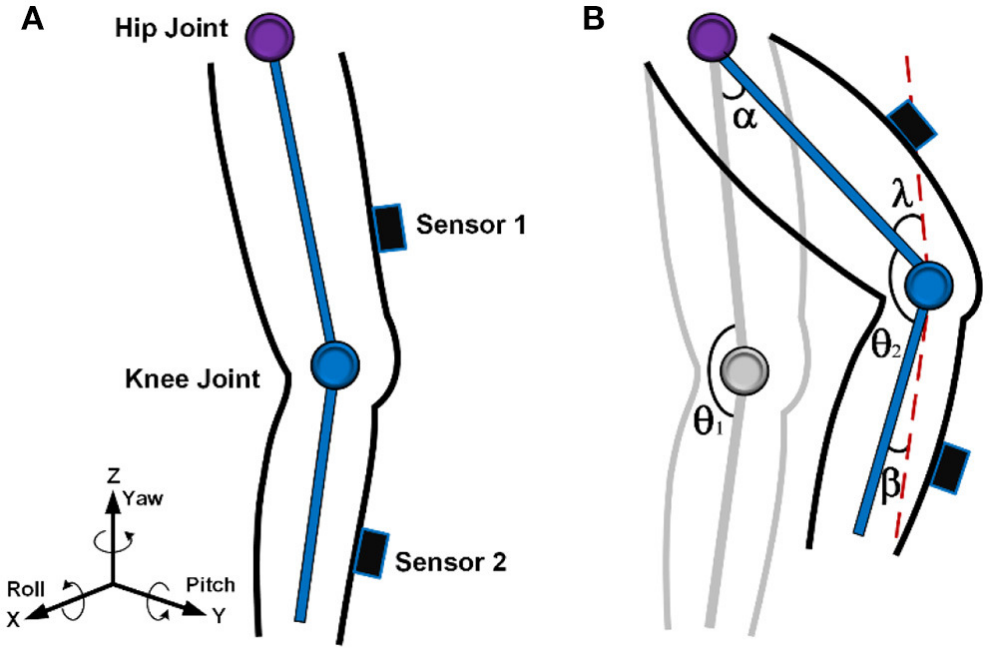
Sensor attitude angle and knee ROM estimation. **(A)** Three-axis acceleration and gyroscope direction. **(B)** Equivalent model of flexion-extension activity.

Before performing data acquisition, the zero drift of the accelerometer needs to be calibrated, otherwise, the data analysis will be biased. In this algorithm, the accelerometer can be auto-zeroed by adding a bias. Figure 4A shows the coordinate axis directions of the three-axis accelerometer. The gyroscope can sequentially integrate the angular velocities around the X, Y, and Z-axis rotations to obtain pitch (θ), roll (γ), and yaw (ψ) angles, respectively.

To describe the attitude angle, a geographic coordinate system n and an object coordinate system b are established. In the static state, the quaternion (q0, q1, q2, q3) is used to describe the rotation information of the three-dimensional space, and the complementary filtering algorithm fuses the data of the accelerometer and the electronic compass, and then updates the quaternion to obtain the latest rotation matrix. In addition, the rotation relationship between coordinate systems can be described by Euler angles, and the rotation matrices from n to b coordinate systems represented by quaternions and Euler angles are expressed as formulas (1) and (2), respectively.


(1) $$\begin{array}{l} C_{n}^{b}=\left[\begin{array}{ccc} q0^{2}+q1^{2}-q2^{2}-q3^{2} & 2\left(q1q2+q0q3\right) & 2\left(q1q3-q0q2\right)\\ 2\left(q1q2-q0q3\right) & q0^{2}-q1^{2}+q2^{2}-q3^{2} & 2\left(q2q3+q0q1\right)\\ 2\left(q1q3+q0q2\right) & 2\left(q2q3-q0q1\right) & q0^{2}-q1^{2}-q2^{2}+q3^{2} \end{array}\right] \end{array}$$

(2) $$\begin{array}{l} C_{n}^{b}=\left[\begin{array}{ccc} \cos\theta \cos\psi & \cos\theta \sin\psi & -\sin\theta \\ -\cos\gamma \sin\psi +\sin\gamma \sin\theta \cos\psi & \cos\gamma \cos\psi +\sin\gamma \sin\theta \sin\psi & \sin\gamma \cos\theta \\ \sin\gamma \sin\psi +\cos\gamma \sin\theta \cos\psi & -\sin\gamma \cos\psi +\cos\gamma \sin\theta \sin\psi & \cos\gamma \cos\theta \end{array}\right] \end{array}$$

Combining the elements between Equations (1) and (2), the attitude angles γ and ψ can be estimated. Expressed as Equations (3) and (4):


(3) $$\begin{array}{l} \theta =-\arcsin\left(2\cdot \left(q_{1}q_{3}-q_{0}q_{2}\right)\right) \end{array}$$

(4) $$\begin{array}{l} \gamma =\arctan\left(\frac{2\cdot \left(q_{2}q_{3}+q_{0}q_{1}\right)}{q_{0}^{2}-q_{1}^{2}-q_{2}^{2}+q_{3}^{2}}\right) \end{array}$$

The complementary filter is a low-complexity filter with strong anti-interference capability commonly used for attitude algorithm, which is to use the angle value obtained by the gyroscope as the optimal value in a short period and to correct the angle obtained by the gyroscope by averaging the acceleration value sampled by the accelerometer at regular intervals (Chang-Siu et al., 2011; Kubelka and Reinstein, 2012). The gyroscope is mainly used in a short time, and the accelerometer is more accurate in a long time. However, accelerometers are susceptible to long-term artifacts, which can cause very noisy attitude angle estimates. We can eliminate the interference caused by artifacts by Equation (5):


(5) $$\begin{array}{l} \left[\begin{array}{c} ex\\ ey\\ ez \end{array}\right]=\left(\frac{1}{\left|1-|f-1|\right|}\right)•\left[\begin{array}{c} ax\\ ay\\ az \end{array}\right]\times \left[\begin{array}{c} vx\\ vy\\ vz \end{array}\right] \end{array}$$

Equation (5) shows that when the accelerometer is subjected to motion artifacts over a longer period, the weight of the difference between the predicted acceleration value and the actual acceleration value can be attenuated to eliminate the oscillatory disturbances brought about by external sources.

*f* is the three-axis combined acceleration, expressed as Equation (6):


(6) $$\begin{array}{l} f=\frac{\sqrt{ax^{2}\cdot ay^{2}\cdot az^{2}}}{g} \end{array}$$

[*vx vy vz*]^T^ is the quaternion representation of g in the b-coordinate system, expressed as Equation (7):


(7) $$\begin{array}{l} \left[\begin{array}{c} vx\\ vy\\ vz \end{array}\right]=\left[\begin{array}{c} 2\cdot \left(q_{1}q_{3}-q_{0}q_{2}\right)\\ 2\cdot \left(q_{2}q_{3}+q_{0}q_{1}\right)\\ g\left(1-2\cdot \left(q_{1}^{2}+q_{2}^{2}\right)\right) \end{array}\right] \end{array}$$

When the error is corrected, the artifacts are eliminated, the quaternion rotation matrix is updated, and finally, a more accurate attitude angle can be obtained.

During the flexion-extension exercise, the patient's knee ROM will change over time, and the swing model is shown in Figure 4B. When sensor 1 is worn on the thigh and sensor 2 is worn on the shank, the ROM is mainly determined by the value of sensor 2, so many studies only estimate the ROM by the swing angle of the shank (Ajdaroski et al., 2020). Although the mobility of the thigh is small, it is necessary to consider the swing angle. The joint angle at the initial stage is denoted as θ_1_, which can be distributed in the range of, for example, 45°-180°. The movement angles of the thigh and shank that swing to the next position are recorded as α and β, respectively. The derivation process of knee ROM between two positions is described as follows.


(8) $$\begin{array}{l} \alpha =\lambda \end{array}$$

(9) $$\begin{array}{l} \theta_{1}=\theta_{2}+\lambda +\beta \end{array}$$

(10) $$\begin{array}{l} ROM=\theta_{1}-\theta_{2}=\alpha +\beta \end{array}$$

Where 1 represents the initial joint angle, and 2 represents the final joint angle; α is the movement angle of the thigh, and β is the movement angle of the shank.

### Data Analysis

In this study, we perform a correlation analysis on the knee ROM recorded from TKR patients and healthy adults. The monitoring and evaluation functions of the wearable inertia system proposed in this paper are verified from three perspectives. First, the ROM results obtained by our method are compared with the results provided by BTE Primus RS, and the differences of the results at the three angular velocities are described through the Bland-Altman plots. Secondly, the maximum flexion-extension ROM of TKR patients and healthy adults are evaluated, and the results of all 20 subjects are displayed by box plots. Finally, we also compare the average knee ROM during gait walking between patients and healthy subjects, which is of great significance for assessing the rehabilitation progress of patients. All experimental results will be presented in detail in Section Result.

## Results

### Results of Passive Flexion-Extension

Table II shows the difference between the knee ROM estimated by our method and the BTE Primus RS. The average and standard deviation of the error corresponding to each angular velocity are calculated. For patients, the average values of errors corresponding to 15°/s, 30°/s, and 45°/s are 3.22°, 4.94°, and 4.34°, respectively. For healthy adults, the average errors of 15°/s, 30°/s, and 45°/s are 0.76°, 0.63°, and 0.16°, respectively. The standard deviations of all subjects at different angular velocities are similar. It can be observed that healthy adults have excellent estimation results, which are better than TKR patients. Due to the pain of TKR patients, their thighs are not tightly fixed on the chair, which is accompanied by swinging during the flexion-extension experiment. Therefore, the error of the patient parameter estimation is enlarged. Even so, from an orthopedic point of view, the ROM estimation results of TKR patients with an average error of less than 5° are clinically acceptable (Huang et al., 2020).

**Figure 5:**
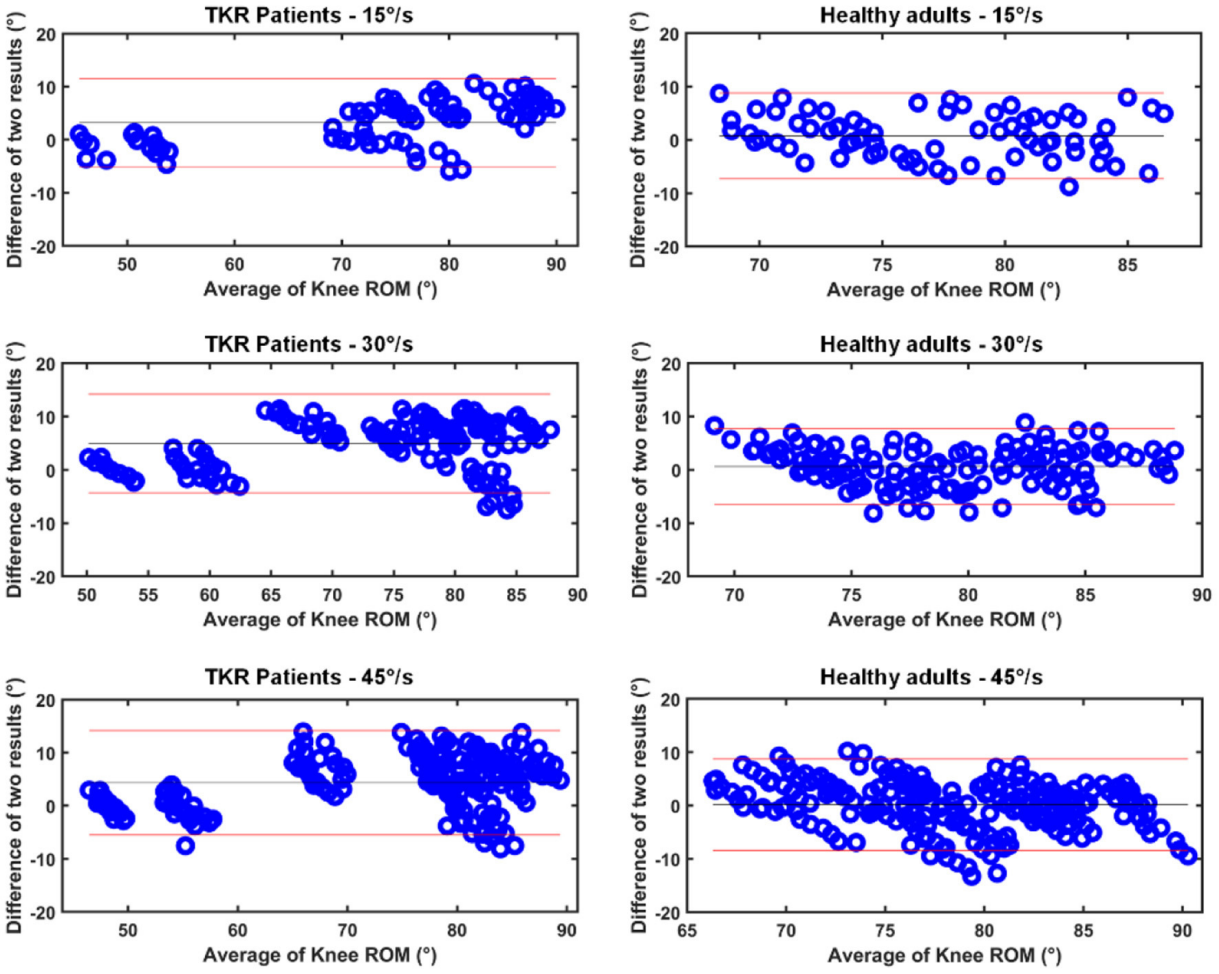
Bland-Altman plot of knee ROM results for two systems. The horizontal axis is the average of the estimates and the vertical axis is the absolute error of the two results.

The Bland-Altman plots of the flexion-extension measurement between the two results are presented (Figure 5). It can be seen that the absolute difference of most flexion and extension activities is within 10°, which is in good agreement with the BTE Primus RS standard. The ROM of healthy adults is concentrated in the range of 70°-90°, which shows the similarity between healthy subjects. The knee ROM of patients is distributed in the range of 45°-90°, which reflects the difference in the recovery level of different subjects. For the same type of subjects, the average error at the three angular velocities does not show an obvious difference, i.e., the angular velocity does not significantly affect the resulting error. In summary, the results of Table 2 and the Bland-Altman plots prove that our ROM estimation method is accurate and reliable.

**Table 2:** The mean and standard deviation (SD) of the difference between IMU sensors and BTE Primus RS estimation results.

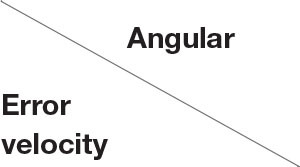	**TKR patients**		**Healthy adults**	
	**Mean (**°**)**	**SD**	**Mean (**°**)**	**SD**
15°/s	3.22	4.23	0.76	4.10
30°/s	4.94	4.72	0.63	3.62
45°/s	4.34	5.02	0.16	4.37

### Results of Active Flexion-Extension

**Figure 6:**
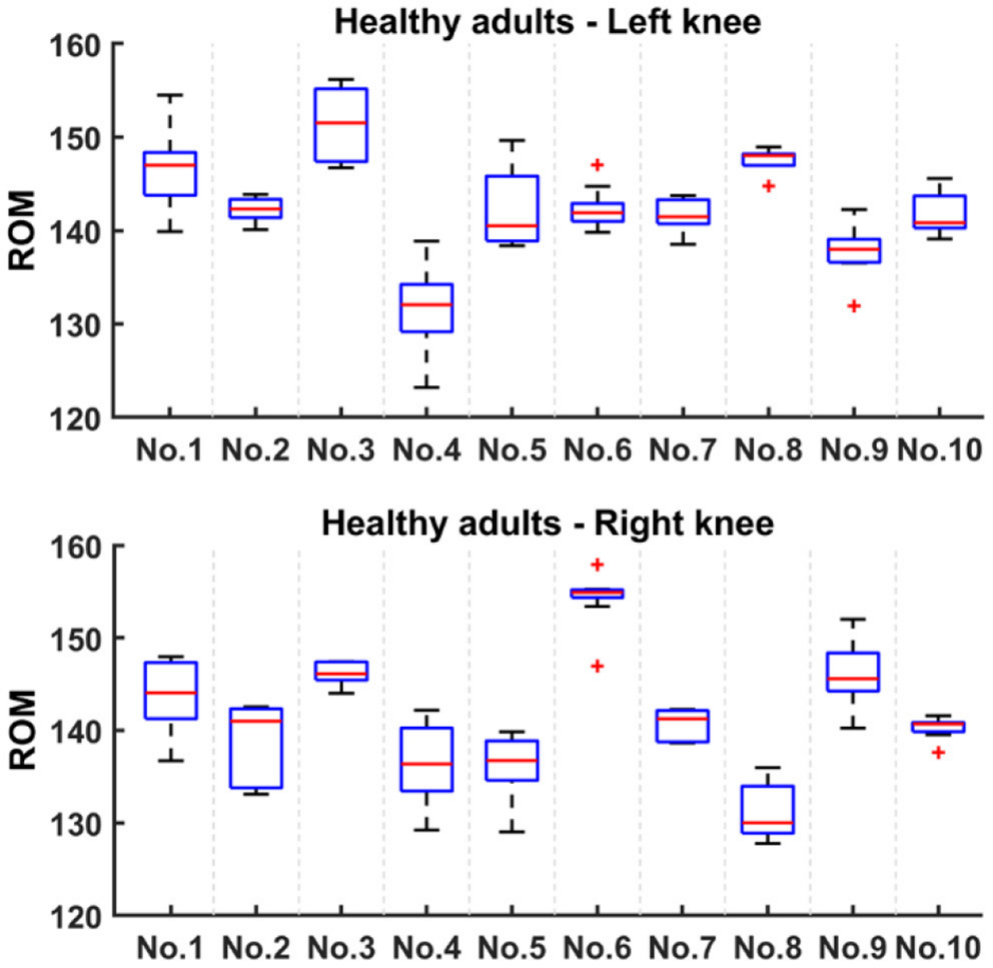
Knee ROM of healthy subjects. They are divided into left and right knee.

The maximum knee ROM of healthy subjects is described in Figure 6. Generally speaking, the difference in knee ROM reflects the level of symmetry on the left-right sides. It can be seen that the knee ROM of each healthy subject is similar, and the difference between left and right ROM is concentrated within 10°, showing good left-right symmetry. Although there are healthy subjects with a difference of more than 10° (e.g., No. 8), it seems reasonable because of individuality and experimental operations.

**Figure 7:**
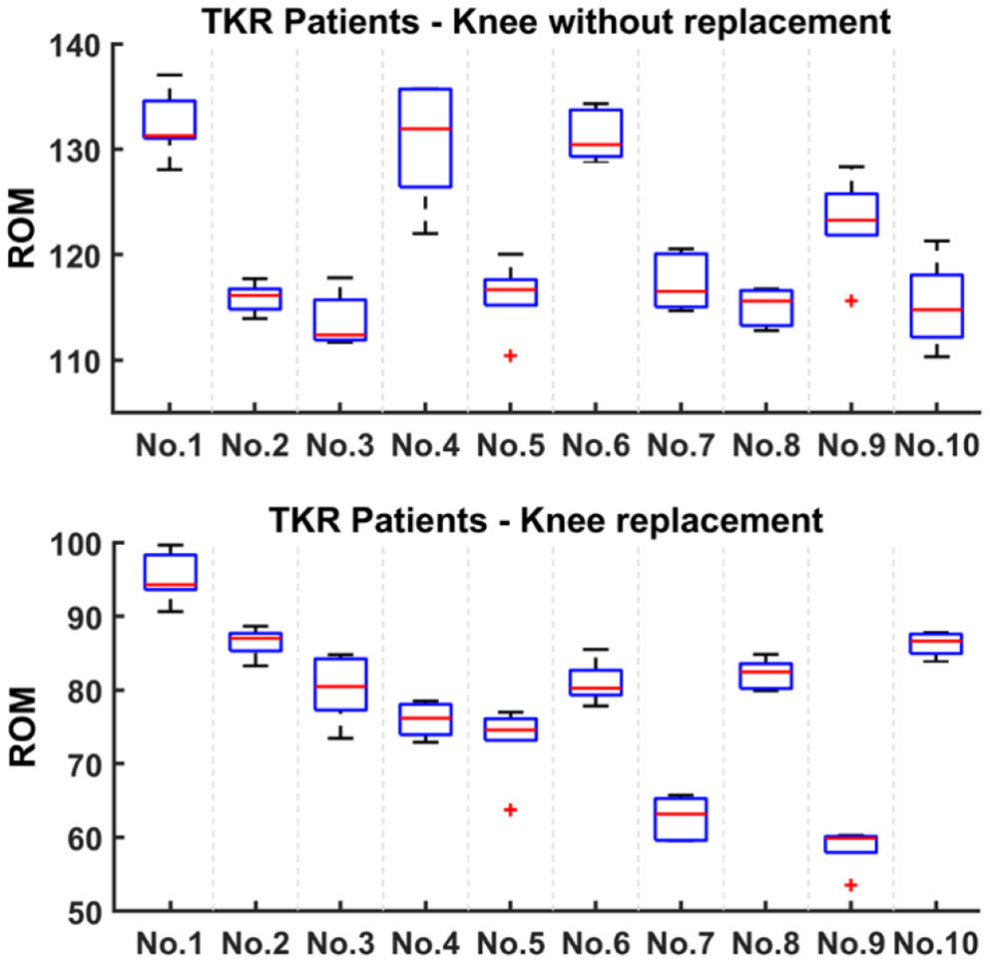
Knee ROM of TKR patients. They are divided into knee replacement and knee without replacement.

Unlike the left-right knee ROM of healthy subjects, TKR patients are shown separately with replacement and without replacement, and the maximum knee ROM of TKR patients is depicted in Figure 7. The difference in knee ROM can also be used to evaluate the patient's rehabilitation progress, i.e., the greater the gap between the two sides, the worse the rehabilitation. Figure 7 shows that the ROM difference between the knees of 10 patients is greater than 30° and even reaches 60° (e.g., No. 9), indicating poor knee function recovery.

### Results of Gait Flexion-Extension

**Figure 8:**
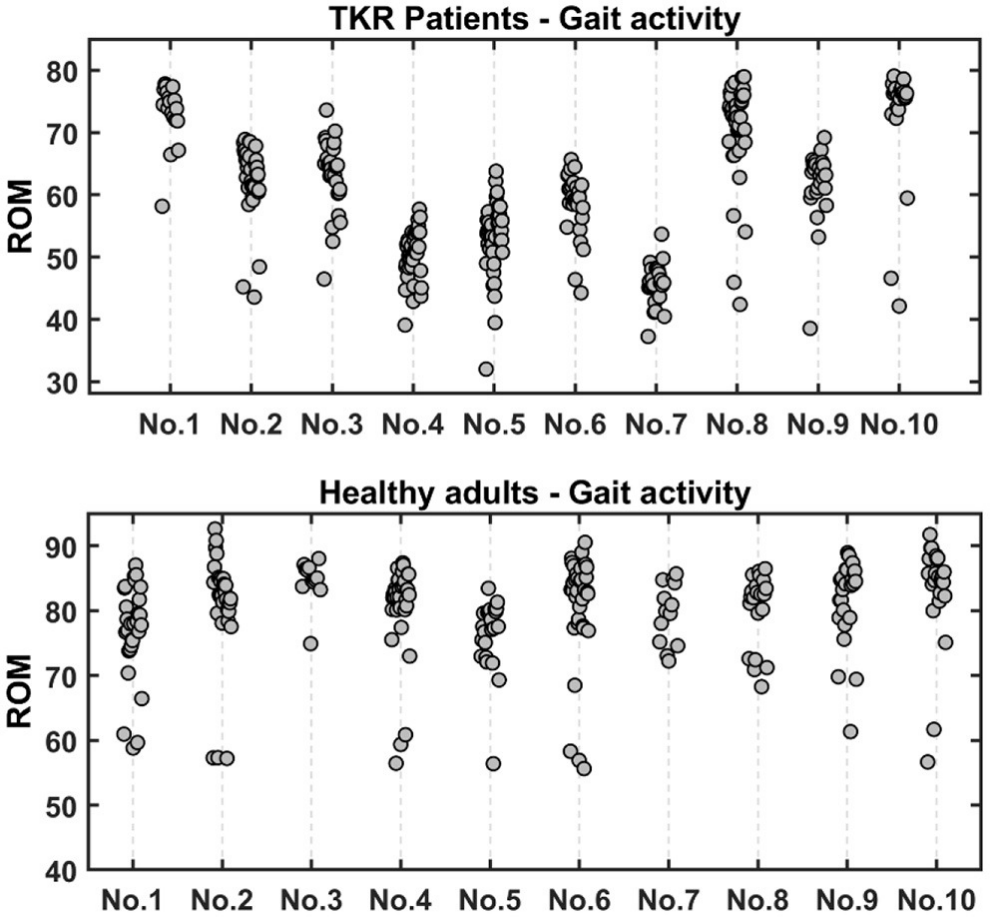
The average knee ROM of gait walking in healthy subjects and TKR patients.

Figure 8 shows the average knee ROM of healthy subjects and TKR patients during gait walking. The knee ROM of walking can reflect the recovery performance of the patient during rehabilitation. It can be seen that the knee ROM of 10 healthy subjects is distributed between 70° and 90°, and the median is close to 80°. The above results indicate that the knee ROM of normal walking should not be less than 70°, which is the same as described in the work of Winemaker et al. (2012). Furthermore, when the patient's walking average knee ROM is concentrated at 80°, it means the knee function returns to normal.

Due to differences in personal conditions and environments, the ROM distribution of the knees of 10 TKR patients is scattered and can be divided into three layers. First, the average knee ROM of 4 patients is less than 60°, indicating poor recovery progress. Secondly, the average knee ROM of the 3 patients ranged from 60° to 70°, which indicates a good recovery. Finally, there are 3 patients with the best recovery whose knee ROM is distributed between 70° and 80°, almost reaching the level of healthy subjects. We compared the recorded videos and found that all the results are consistent with the video performance.

## Discussion

In this work, we developed a portable and wearable IMU software and hardware platform and designed a systematic monitoring and evaluation program to address the clinical needs of TKR patients after surgery. Through dynamic and static flexion and extension activities, the accuracy and practicability of the system are verified. The main contributions of this research include: (1) The developed software and hardware platform is portable and reliable and can be used by patients at home without being restricted by time and space; (2) The system performed a series of comprehensive flexion and extension experimental verification on TKR patients and healthy adults, which is more detailed than previous studies; (3) The proposed ROM estimation algorithm has higher accuracy and can solve the problem of sensor zero drift and motion artifacts. The specific contributions of this paper are discussed in detail as follows.

Although some studies have estimated the knee ROM in patients with TKR (Mcginnis et al., 2016; Bell et al., 2019), the research focusing on monitoring the ROM of the knee joint is relatively insufficient. In addition, there are still some shortcomings in the system verification and estimation methods, which limit the real home application of the wearable system. Chen et al. (2015) designed an assessment method for knee osteoarthritis rehabilitation exercise, which showed good results in healthy people, but it was not confirmed by patient data. Huang et al. (2020) monitored the knee ROM of healthy people and patients and showed high accuracy, but there is a lack of monitoring of the rehabilitation progress of patients during gait walking. However, the ROM of gait activity, as an important indicator of knee joint function, is particularly important for the evaluation results (Lee et al., 2018; Blakeney et al., 2019). In addition, many rehabilitation assessment methods only use the knee ROM of active flexion-extension activity as the evaluation index (Chiang et al., 2017). Overall, to complete the monitoring and evaluation of knee ROM more accurately and carefully, we subdivided the experiment into active and passive flexion-extension activities and gait activities.

In Section Results of Passive Flexion-Extension, the knee ROM estimated by our proposed algorithm and professional equipment BTE Primus RS is described in the Bland-Altman plots. The average error of all subjects is in the range of 0.16° to 4.94°, which is consistent with related studies reported previously (Bakhshi et al., 2011; Feldhege et al., 2015; Chiang et al., 2017; Ajdaroski et al., 2020). Specifically, Chiang et al. (2017) monitored the movement of patients with TKR, but the zero-drift problem of the system requires a professional operation, and the automatic calibration of the system has certain advantages. Ajdaroski et al. (2020) verified the performance of a single sensor for dynamic knee angle measurement, and the results showed that the absolute average difference in flexion/extension measurement between the two systems was 8.43°, which was worse than our result due to the problem of motion artifacts. Feldhege et al. (2015) estimated the knee ROM of healthy subjects and patients with multiple sclerosis, and the results showed that the root means the square error was less than 5°. Although the accuracy is comparable to our results, the proposed algorithm needs to measure parameters such as the distance between the sensor and the joint, which is difficult to generalize to clinical use. Bakhshi et al. (2011) reported an average error range of 0.08° to 3.06°, but this method has only been verified on a healthy subject, and its performance in TKR patient needs further explanation. A state-of-the-art comparison of knee ROM estimation is shown in Table 3. In summary, our proposed system has functions comparable to professional equipment and has the higher potential to monitor rehabilitation training.

**Table 3:** The comparison of state-of-art on knee ROM estimation.

**References**	**Subjects**	**Activities**	**ROM average error (**°**)**	**Motion artifacts**
Chiang et al. (2017)	18 TKR patients	Active flexion and extension; gait activities	**/**	Y
Bell et al. (2019)	10 healthy adults	Heel slides; Short arc quadriceps; Sit-to-stand	2.4°; 2.0°; 2.9°	Y
Ajdaroski et al. (2020)	8 healthy adults	Jump activity	8.11°	Y
Huang et al. (2020)	16 TKR patients 8 healthy adults	Passive flexion and extension	Healthy: 2.90°; 3.51°; 4.00°; Patients: 1.65°; 2.74°; 3.27°	Y
Our study (2021)	10 TKR patients 10 healthy adults	Active/ Passive flexion and extension; Gait activities	Healthy: 3.22°; 4.94°; 4.34°; Patients: 0.76°; 0.63°; 0.16°	N

To verify that ROM can be used as an evaluation indicator of knee function recovery, we made a series of analyses and comparisons in Section Results of Active Flexion-Extension and Results of Gait Flexion-Extension. In general, the smaller the ROM difference between the left and right knees, the better the knee joint recovery. In Section Results of Active Flexion-Extension, we found that the difference in ROM between left and right knees of healthy subjects was much smaller than that of patients, i.e., healthy subjects had better left-right symmetry. In Section Results of Gait Flexion-Extension, the ROM of gait flexion-extension activity is described. Through the average ROM of gait activity, the recovery level of patients can be well-identified. It turns out that knee ROM is effective for knee joint recovery assessment, which is in keeping with previous studies (Winemaker et al., 2012; Issa et al., 2015; Ramkumar et al., 2019). Because different activities require different ROMs (Chiang et al., 2017), our goal is to perform rehabilitation monitoring and evaluation on patients through the constructed system to increase their maximum flexion-extension ROM to at least 115°, while the average gait ROM is not less than 80°.

On the whole, the proposed sensor system achieves the three expected goals well. Due to its simple operation and high execution efficiency, the system has strong clinical application value, which can provide assessment for patients and provide assistant to physical therapists. In addition, the wearable inertial system has good social benefits and advantages. It can be free from time-space constraints, convenient for patients to use at home, and save medical costs and hospital resources. We anticipate the system proposed in this paper can be used for long-term monitoring and evaluation of many symptoms, such as TKR, anterior cruciate ligament (ACL), and fractures, etc. Furthermore, an active rehabilitation scheme can be developed for patients at home to restore knee function. In the future, the proposed method is expected to be transplanted into the sensor-based control system in combination with machine learning algorithms, which in turn can identify and alert patients of different recovery stages.

We admit that our research has some limitations. First, in evaluating the rehabilitation progress of TKR patients, we uniformly screened patients 1 week after surgery. Although we have made rich comparisons between healthy people and patients, and the knee replacement and non-replacement side of the patient, there is still a lack of comparisons between patients on the surgical site in different periods. For example, the comparison of the time before the operation and 1 week, 2 weeks, or 1 month after the operation (Ramkumar et al., 2019). Secondly, we only estimated the knee ROM parameter. Although we have confirmed that knee ROM is a good predictor of knee functional recovery, to better monitor the rehabilitation exercise, other parameters such as the number and frequency of flexion can be estimated (Chapman et al., 2019; Huang et al., 2020). Fortunately, new patients with osteoarthritis have been recruited, and we have planned a 3-month follow-up before and after surgery, which will further verify the clinical value of the system proposed in this paper. We have reason to believe in the good performance of the system.

## Conclusion

In the work, we proved that our proposed system can be comparable to professional equipment BTE Primus RS. In addition, the constructed monitoring and evaluation system has also been proven to accurately assess the rehabilitation level of patients. The results demonstrate that knee ROM is of great significance as a key indicator for assessing patient knee function recovery. In the future, we hope this system can provide long-term effective supervision of patients in clinical and dwelling environments.

## Data Availability Statement

The original contributions presented in the study are included in the article/supplementary material, further inquiries can be directed to the corresponding author/s.

## Ethics Statement

The studies involving human participants were reviewed and approved by the Medical Research Ethics Committee of the Hong Kong University Shenzhen Hospital [No. Lun (2019) 175]. The patients/participants provided their written informed consent to participate in this study. The animal study was reviewed and approved by the Medical Research Ethics Committee of the Hong Kong University Shenzhen Hospital [No. Lun (2019) 175]. Written informed consent was obtained from the individual(s) for the publication of any potentially identifiable images or data included in this article.

## Author Contributions

NL and YD contributed to the conception and design of the study and wrote the first draft of the manuscript. QC, YN, SL, and GaL conducted experiments. YD and QC analysed the data. YN contributed to the development of sensors. GZ and GuL contributed to manuscript revision. All authors have read and approved the submitted manuscript.

## Funding

This study has been financed partially by the National Key R&D Program of China (2018YFC2001400/04, 2019YFB1311400/01), the Shandong Key R&D Program (2019JZZY011112), the Innovation Talent Fund of Guangdong Tezhi Plan (2019TQ05Z735), the High Level-Hospital Program, Health Commission of Guangdong Province (HKUSZH201901023), and the Guangdong-Hong Kong-Macao Joint Laboratory of Human-Machine Intelligence-Synergy Systems (2019B121205007).

## Conflict of Interest

The authors declare that the research was conducted in the absence of any commercial or financial relationships that could be construed as a potential conflict of interest.

## Publisher's Note

All claims expressed in this article are solely those of the authors and do not necessarily represent those of their affiliated organizations, or those of the publisher, the editors and the reviewers. Any product that may be evaluated in this article, or claim that may be made by its manufacturer, is not guaranteed or endorsed by the publisher.
